# A Cost-Effective Task Trainer for Complex Lip Laceration Repair

**DOI:** 10.7759/cureus.13659

**Published:** 2021-03-02

**Authors:** Ryan Walsh, Charles Lei, Jeffrey Heimiller, Joseph Sikon, Kenneth H Palm

**Affiliations:** 1 Department of Emergency Medicine, Vanderbilt University Medical Center, Nashville, USA

**Keywords:** task-trainer, vermillion border, complex lip laceration repair, emergency medicine procedural competency, emergency medicine training, laceration repair, emergency medicine

## Abstract

Facial laceration repair is a common emergency department procedure with important cosmetic implications for patients. In instances where the vermillion border is violated special attention must be paid to accurate opposition, as little as 1 mm of misalignment can result in poor cosmetic results. We sought to construct and evaluate an affordable, effective, and easily reproduced simulation trainer of full-thickness lip laceration requiring vermillion border repair primarily for Emergency Medicine resident education.

To accomplish this we utilized microfoam tape, 4x4 gauze, self-adherent wrap, and markers to simulate a multi-layered lip laceration with vermillion border involvement. The microfoam tape with gauze folded on top of itself simulates the orbicularis oris muscle and subcutaneous fat layer. The self-adherent gauze covered by an additional piece of microfoam tape simulates the dermal/epidermal junction. This training model can be attached to an upside-down emesis basin with tape and then trainees can practice appropriate repair techniques.

This task trainer was then utilized in our scheduled, simulation didactic sessions with Vanderbilt University Medical Center’s Emergency Medicine residents. In total, 23 PGY 1-3 EM residents participate in the session. Nineteen (83%) completed an anonymous reporting survey rating features of the didactic on a five-point Likert scale. Resident comfort level performing the procedure prior to the teaching session was fair (mean 2.53 {SD 1.04}) and afterward significantly higher (mean 4.31 {SD 0.57}) P <0.0001. The task trainer was highly rated (mean 4.74 {SD 0.55}) and the overall didactic was also very highly rated (mean 4.84 {SD 0.50}).

The model we have described here can be constructed in minutes from supplies that are readily available in any healthcare setting and was rated by residents to substantially improve procedural confidence in regards to complex lip laceration repair.

## Introduction

Facial laceration repair is a common emergency department procedure. The repair of complex lip lacerations requires particular attention to detail in order to maintain satisfactory functional and cosmetic outcomes.

Large, full-thickness lip lacerations typically require a multi-layered closure. This strategy preserves orbicularis oris function and allows for a lower-tension superficial closure to maximize cosmetic outcomes [1]. Attention must especially be paid to the apposition of the vermillion border, whereas little as 1 mm of misalignment may result in unacceptable cosmetic results [2]. Because of the high stakes of lip laceration repair, novice learners may appreciate the opportunity to first practice this procedure on a task trainer prior to live application. While multi-layered wound closure models are available commercially and others have created their own models using readily available hospital supplies, we are unaware of any published models that simulate a complex lip laceration [3].

We developed a low-cost, easily reproduced simulation model to train emergency medicine residents to repair full-thickness lip lacerations involving the vermillion border. We hypothesized that the model would significantly improve resident comfort in performing this procedure.

## Technical report

Materials

The model was made from supplies readily available in most emergency departments (Table 1). Utilizing these supplies as described below, we were able to create multiple models in a very short period of time. These supplies also allowed for easy reproducibility of creating the training model. 

**Table 1:** Materials to create the model

Table 1
4-inch 3M white microfoam tape
4-inch by 4-inch gauze
Self-adherent wrap
Red marker
Scissors
Emesis basin

Model creation

**Figure 1:**
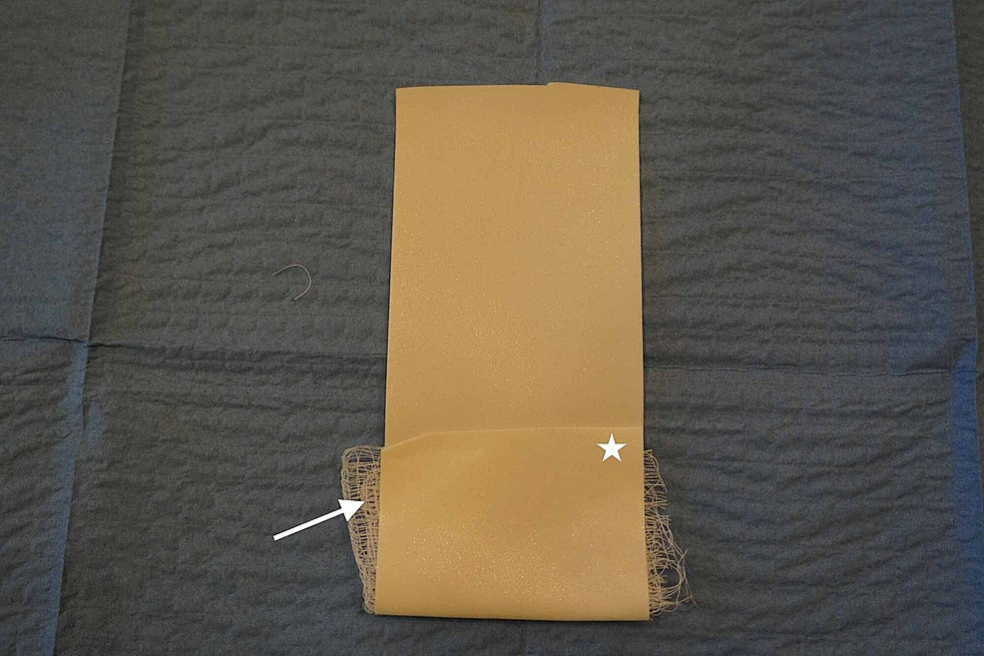
First steps in the creation of the model Star: microfoam tape folded Arrow: one layer of 4-inch x 4-inch gauze

The first step in the creation of the model was to unroll 6 inches in length of microfoam tape with the non-adherent side facing the table. We then applied one layer of 4-inch by 4-inch gauze at one end of the adherent side of the tape. Next, we folded the microfoam tape and gauze over itself (Figure 1). 

**Figure 2:**
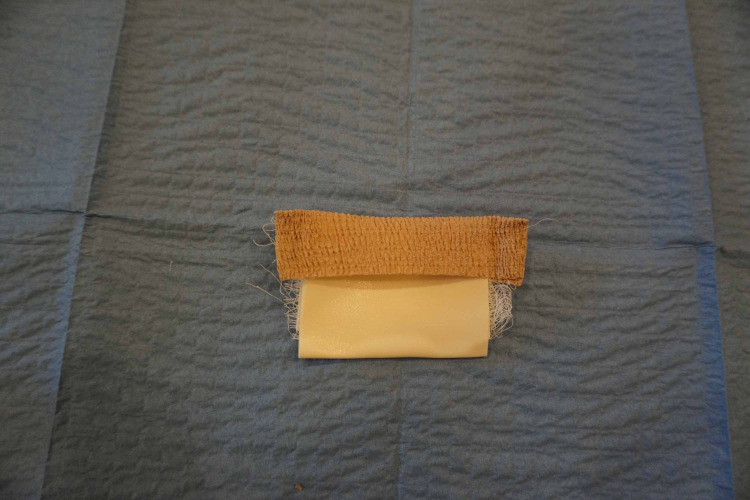
Microfoam tape folded on top of itself with self-adherent wrap over one edge

We continued to fold the tape over on itself until the piece was approximately 3 inches in length. We then wrapped a piece of the self-adherent wrap over the top of the foam tape (Figure 2). 

**Figure 3:**
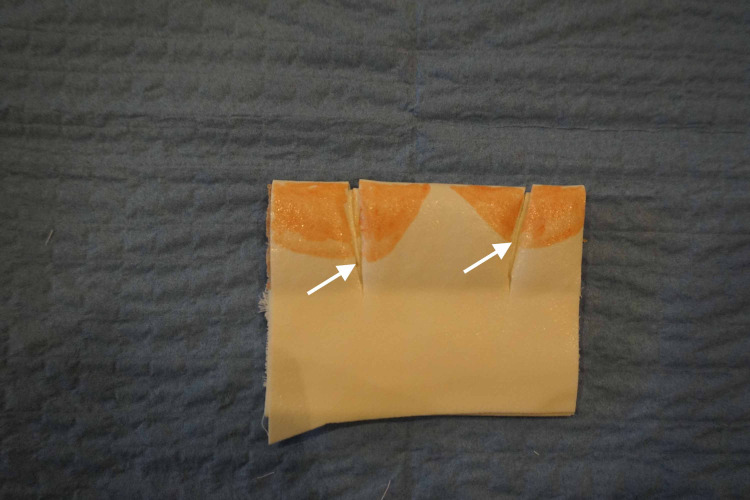
A pair of lips is drawn on the microfoam tape with a simulated through and through the laceration Arrow: vermillion border

We covered the self-adherent wrap with another piece of microfoam tape and used the red marker to draw two sets of lower lips. We then used the scissors to create a laceration long enough to traverse the simulated “vermillion border” (Figure 3). 

**Figure 4:**
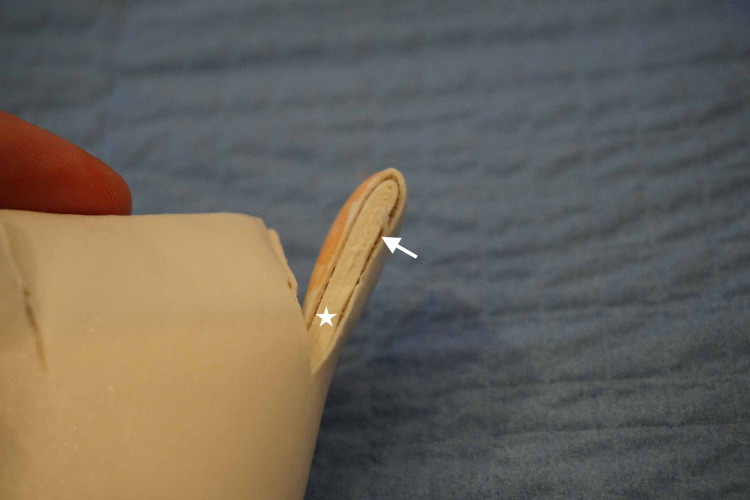
A side view of the model with the microfoam tape folded on itself Star: simulated orbicularis oris muscle and subcutaneous fat Arrow: self-adherent wrap covered by additional microfoam simulating the dermal/epidermal junction

The microfoam tape with gauze folded on top of itself simulates the orbicularis oris muscle and subcutaneous fat layer. The self-adherent wrap covered by an additional piece of microfoam tape simulates the dermal/epidermal junction (Figure 4). 

**Figure 5:**
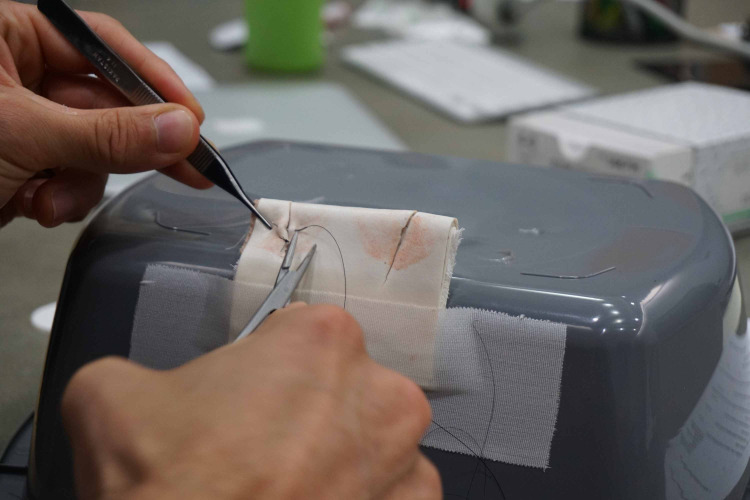
Completed task trainer being utilized by a learner

This training model can be attached to an upside-down emesis basin with tape and then both deep and superficial sutures can be placed in the simulated lips (Figure 5).

Results

A total of 23 PGY 1-3 emergency medicine residents participated in the complex lip laceration repair procedural session. Eighty-three percent (19/23) of the residents completed anonymous post-course surveys in which they rated aspects of the session on a five-point Likert scale. Both the overall teaching session (mean 4.84, SD 0.50) and the task trainer (mean 4.74, SD 0.55) were rated very highly. A resident commented that a significant strength of the task trainer was that it simulated an orbicularis oris closure well and another resident stated that “the model represents the layers effectively.” Compared to pre-course ratings, residents reported statistically significant post-course improvements in their level of confidence in performing the procedure (mean 2.53 {SD1.04} versus mean 4.31 {SD 0.57}; P <0.0001].

## Discussion

Wound closure is an important procedural skill that is critical to the practice of emergency medicine [4]. We have developed a complex lip laceration repair model that is cost-effective and easy to build. The task trainer was well-received by a group of emergency medicine residents, who noted that it effectively simulated the anatomy of the lip and increased their confidence in performing the procedure.

Many programs have significant financial constraints on purchasing commercially available procedural training equipment. A number of homemade wound closure models have been described in the literature [5,6]. While relatively inexpensive, these models often require a substantial time commitment to procure the necessary supplies and complete the building process. The model we have described here can be constructed in minutes from supplies that are readily available in any healthcare setting. This allows learners to receive just-in-time training on repairing a complex lip laceration immediately before performing the procedure on an actual patient [7].

Our participant group was relatively small and consisted solely of emergency medicine residents. In addition, we only evaluated participant reactions to the model. Further research is needed to determine if training using this model leads to improved wound closure skills and patient outcomes.

## Conclusions

We have created a low-cost, reproducible model for training complex lip laceration repair. We hope that other emergency medicine residencies and surgical training programs will find this model valuable.

## References

[1] Grunebaum L, Smith J, Hoosien G (2010). Lip and perioral trauma. Facial Plastic Surg.

[2] Heintz W (1977). Traumatic injuries: dealing with dental injuries. Postgrad Med.

[3] (2019). A cost-effective, two-layer wound closure task trainer. https://www.aliem.com/2015/04/cost-effective-two-layer-wound-closure-task-trainer/.

[4] Counselman FL, Babu K, Edens MA (2017). The 2016 model of the clinical practice of emergency medicine. J Emerg Med.

[5] Rahmani G, McArdle A, Kelly JL (2016). A quick and easy makeshift suture pad. Med Educ.

[6] Weeks D, Kasdan ML, Wilhelmi BJ (2015). An inexpensive suture practice board. Eplasty.

[7] Niles D, Sutton RM, Donoghue A (2009). “Rolling refreshers”: a novel approach to maintain CPR psychomotor skill competence. Resuscitation.

